# Detection of serum VEGF and MMP-9 levels by Luminex multiplexed assays in patients with breast infiltrative ductal carcinoma

**DOI:** 10.3892/etm.2014.1685

**Published:** 2014-04-14

**Authors:** JUNYING ZHANG, LI YIN, JIANZHONG WU, YE ZHANG, TAO XU, RONG MA, HAIXIA CAO, JINHAI TANG

**Affiliations:** 1 Department of Oncology, Xuzhou Medical College, Xuzhou, Jiangsu 221004, P.R. China; 2 Department of Radiation Oncology, Nanjing Medical University Affiliated Cancer Hospital, Jiangsu Cancer Hospital and Jiangsu Institute of Cancer Research, Nanjing, Jiangsu 210009, P.R. China; 3 Research Center for Clinical Oncology, Nanjing Medical University Affiliated Cancer Hospital, Jiangsu Cancer Hospital and Jiangsu Institute of Cancer Research, Nanjing, Jiangsu 210009, P.R. China; 4 Department of General Surgery, Nanjing Medical University Affiliated Cancer Hospital, Jiangsu Cancer Hospital and Jiangsu Institute of Cancer Research, Nanjing, Jiangsu 210009, P.R. China

**Keywords:** breast infiltrative ductal carcinoma, liquid chip-based method, vascular endothelial growth factor, matrix metalloproteinase-9

## Abstract

The aim of the present study was to assess the effect of the combined detection of serum vascular endothelial growth factor (VEGF) and matrix metalloproteinase-9 (MMP-9) by Luminex multiplexed assays for the diagnosis, treatment and prognosis of breast cancer. Preoperative levels of serum VEGF and MMP-9 were detected via a lipid chip-based method in 301 breast cancer cases, 83 breast fibroadenoma cases and 40 healthy adults. Postoperative levels of VEGF and MMP-9 were also detected in 118 breast cancer cases. The levels of serum VEGF and MMP-9 in patients with breast infiltrative ductal carcinoma (IDC) were higher than those in the breast fibroadenoma and healthy control groups (P<0.05); there was no statistically significant difference between the breast fibroadenoma and healthy groups (P>0.05). The levels of VEGF and MMP-9 were shown to correlate with the clinical stage, tumor size and the lymph node metastasis status. However, the levels were not associated with age or gender (P>0.05). In addition, the serum level of MMP-9 exhibited a significantly correlation with the VEGF level (r=0.601, P<0.001). Subgroup analysis revealed that in patients with IDC, serum levels of VEGF and MMP-9 prior to surgery were significantly higher than those following surgery (P<0.05). Therefore, the serum levels of VEGF and MMP-9 can be used as markers for the diagnosis of breast IDC and may also be valuable for the prediction of lymph nodes metastasis.

## Introduction

Breast cancer is the leading cause of cancer mortality among females in China, and the incidence of breast cancer is increasing continuously. Metastasis is the most important factor that leads to treatment failure and mortality (1). Epithelial-mesenchymal transition (EMT) and vascularization are known to be key factors in tumor invasion and metastasis (2). Vascular endothelial growth factor (VEGF) and matrix metalloproteinases (MMPs) play important roles in EMT. MMPs promote tumor invasion and metastasis in breast cancer, thus, facilitate the departure of epithelial cells from their surrounding tissue. The underlying mechanisms include the degradation of the extracellular matrix and intercellular contacts by MMPs (3). A previous study demonstrated that MMP-9 can accelerate tumor metastasis by promoting neovascularization and lymphangiogenesis (4). VEGF is also considered to be an important and effective factor in stimulating vascularization, which participates in tumor invasion and metastasis (5). A number of studies have described a correlation between VEGF and MMP-9 levels (6). However, the value of combined detection of VEGF and MMP-9 levels in breast cancer remains unclear. Therefore, the aim of the present study was to determine whether the combined detection of VEGF and MMP-9 is valuable for the diagnosis, treatment and prognosis of breast cancer. For this purpose, the levels of VEGF and MMP-9 were compared among breast cancer patients, fibroadenoma patients and healthy adults using a lipid-chip based method.

## Patients and methods

### Patients

A total of 301 breast cancer patients, 83 fibroadenoma patients and 40 healthy adults without breast-associated diseases were enrolled in the study. All the cases were female and had been diagnosed between September 2011 and December 2012 in Jiangsu Province Cancer Hospital (Nanjing, China). Blood samples were collected from the 301 breast cancer patients, of which the postoperative pathology was infiltrative ductal carcinoma (IDC), prior to surgery or any other therapy. Blood samples were also collected postoperatively from 118 of the 301 patients. The 301 breast cancer patients were aged between 27 and 80 years, with a median age of 51 years. The 118 patients who underwent surgery had an age range of 28–80 years and the median age was 51 years. In the fibroadenoma group, the 83 patients were aged between 19 and 58 years, with a median age of 27 years, while the 40 healthy adults had an age range of 19–48 years and a median age of 28 years. According to the Tumor, Node, Metastasis (TNM) staging criteria from the Union for International Cancer Control (7th edition) (7), the 301 breast cancer patients comprised 125 phase I cases, 58 phase II cases, 98 phase III cases and 20 stage IV cases, among which 127 patients were diagnosed with lymph node metastasis, 166 patients were without lymph node metastasis and in eight cases, the status of the lymph node was not detected (without axillary lymph node dissection). All patients provided informed consent. The participants serum was obtained by the Ethics Committee of Jiangsu Cancer Hospital (Nanjing, China) following approval.

### Collection and preservation of the specimens

Venous fasting blood samples (3 ml) were collected and placed in test tubes without endotoxin or pyrogen. The tubes were shaken three times and then left at room temperature for 30 min in order for coagulation to occur. The samples were centrifuged for 10 min at 1,000 × g and the blood serum was extracted and stored in a refrigerator at −80°C for further analysis. Blood samples from the breast cancer and fibroadenoma patients were collected prior to surgery or any other therapy. In the 118 breast cancer patients that underwent surgery, the blood samples were collected one month following surgery.

### Reagents

Test detection was performed using Luminex multiplexed assays. FLEXMAP 3D^®^ system consists was provided by Luminex Corporation (Austin, TX, USA). A Human Cytokine/Chemokine and Human CVD Panel I kit (Millipore, Billerica, MA, USA) was used to detect the levels of serum VEGF and MMP-9.

### Experimental procedures

For the main experimental procedure of VEGF and MMP-9, all the above reagents needed to warm to room temperature (about 20 to 25°C) prior to be used. The placement of standards of VEGF was 0 (background), 3.2, 16, 80, 400, 2,000 and 10,000 pg/ml while of MMP-9 was 0 (background), 0.016, 0.08, 0.4, 2.0, 10.0, 50.0 ng/ml, then controls and test specimens were added to the plate (25 μl per well). The aforementioned procedures were conducted on ice. Next, 25 μl assay buffer was added to each well. The specimens were shaken at room temperature; VEGF for 16 h and MMP-9 for 2 h, ensuring that the procedures were conducted away from the light. Following washing twice, 25 μl detection antibody was respectively added to each well and the plates were shaken for 1 h at room temperature. Next, 50 μl streptavidin-biotin-phycoerythrin medium was added per well and the specimens were shaken again at room temperature for 30 min. Washed them twice with wash buffer and sheath fluid (150 μl for VEGF and 100 μl for MMP-9) with 200 μl per well. Added the lotion to the board after washed it twice. The plates were run with the FLEXMAP 3D™ system and in order to calculate the samples concentration, the median fluorescence intensity results were analyzed by a weighted five-parameter logistic method. Three independent experiments were performed.

### Quantitative detection of the protein levels

It needed warm-up before for 30 min. Instrument parameters were set as follows: Events, 50; gate, 8,000–15,000; sample sizes, VEGF 100 μl and MMP-9 50 μl; time out, 60 sec; bead regions, VEGF 86 and MMP-9 27. The specimens were placed in a detecting pool, running procedures related to the operation. Microspheres median fluorescence readings were analyzed using xPONENT^®^ 4 software (Luminex Corporation) to obtain the final result. Three independent experiments were performed.

### Statistical analysis

Statistical analysis was conducted using SPSS version 20.0 (IBM, Armonk, NY, USA). As statistical analysis revealed the data to have a skewed distribution, non-parametric tests were selected. Comparisons between two groups were performed using the independent samples Mann-Whitney U test, while independent samples Kruskal-Wallis one-way analysis of variance was used for multiple group comparisons. Spearman’s ρ was used to analyze the correlation between two factors, where r≥0.8 was considered to indicate a highly significant correlation, 0.5≤r<0.8 was considered to indicate a moderate correlation, 0.3≤r<0.5 was considered to indicate a low correlation and r<0.3 was considered to indicate a weak or irrelevant correlation. P<0.05 was considered to indicate a statistically significant result.

## Results

### Serum VEGF and MMP-9 levels in the pretreatment groups of IDC, patients with breast fibroadenoma and healthy controls

**Figure 1:**
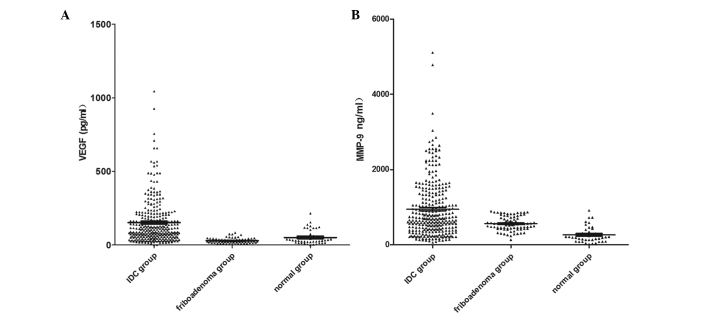
Comparisons in the levels of serum (A) VEGF and (B) MMP-9 among IDC, fibroadenoma and healthy individuals. VEGF, vascular endothelial growth factor; MMP-9, matrix metalloproteinase-9; IDC, infiltrative ductal carcinoma.

Mean levels of serum VEGF and MMP-9 were significantly higher in the breast cancer patients when compared with the levels in the breast fibroadenoma patients and the healthy controls (Table I; Fig. 1).

**Figure 2:**
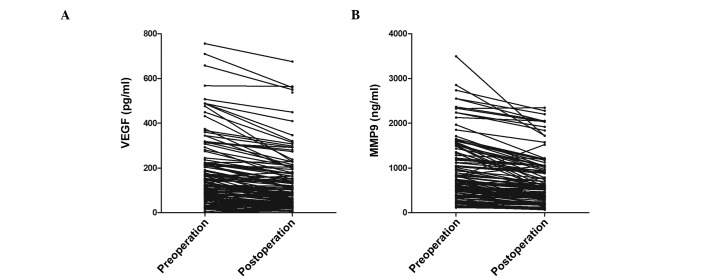
Comparison in the levels of serum (A) VEGF and (B) MMP-9 between preoperative and postoperative patients with IDC. VEGF, vascular endothelial growth factor; MMP-9, matrix metalloproteinase-9; IDC, infiltrative ductal carcinoma.

The pre-operation and post-operation levels of VEGF and MMP-9 were detected respectively. Compared with the pre-operation group, the levels of VEGF and MMP-9 were all significantly decreased following surgery. [VEGF: 180.89±167.82 vs. 135.26±131.20 (P<0.05); MMP-9: 903.92±704.76 vs. 680.36±551.77 (P<0.05)] (Fig. 2).

### Correlation between serum VEGF and MMP-9 levels and clinicopathological parameters in the pretreatment group of IDC analysis

The association between serum VEGF and MMP-9 levels with clinicopathological characteristics in IDC patients (Table II) was analyzed. Serum levels of VEGF and MMP-9 were closely associated with tumor size (P<0.001), and the serum levels in patients with stage III or IV breast cancer were significantly higher than those classified as TNM stage I or II (P<0.001). In addition, the protein levels of VEGF and MMP-9 in patients with lymph node metastasis were significantly higher than those without lymph node metastasis (P<0.001). However, the serum levels of VEGF and MMP-9 exhibited no statistically significant difference between the different age groups in the breast cancer patients (P>0.05).

**Figure 3:**
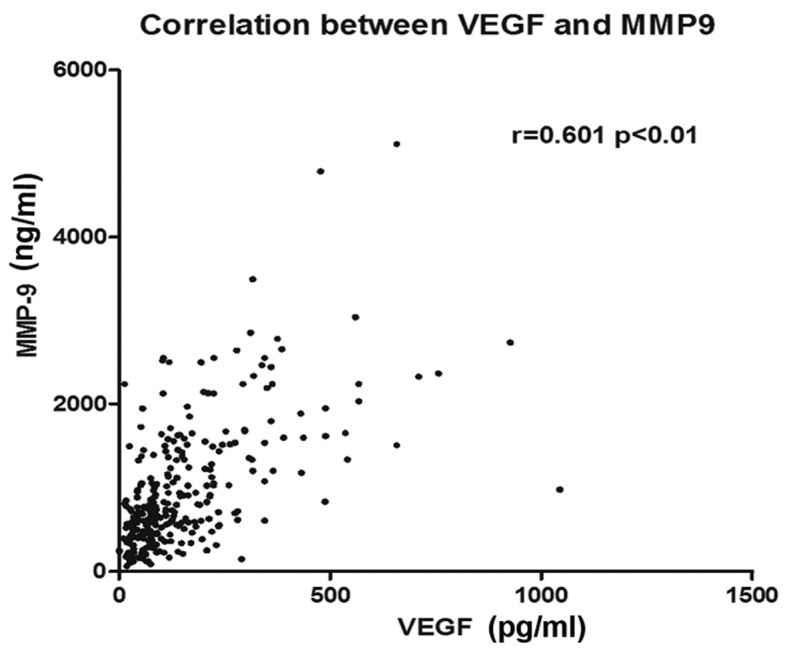
Correlation between serum VEGF and MMP-9 levels in IDC patients. VEGF, vascular endothelial growth factor; MMP-9, matrix metalloproteinase-9; IDC, infiltrative ductal carcinoma.

Spearman’s ρ analysis revealed a significant positive correlation between the serum level of VEGF and serum the level of MMP-9 (Fig. 3; r=0.601, P<0.001).

### Diagnostic value of VEGF and MMP-9 in the pretreatment group of NIDC

**Figure 4:**
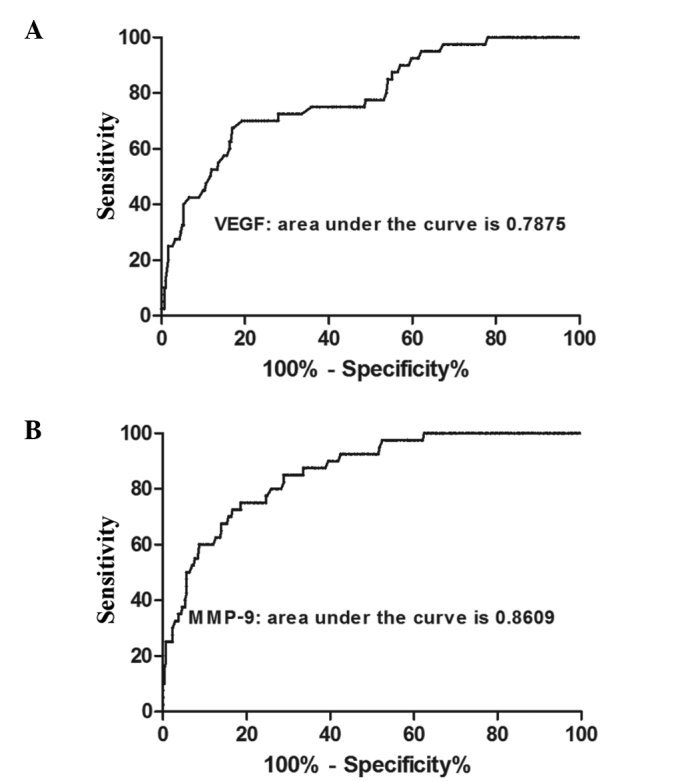
Area under the ROC curves for the levels of serum (A) VEGF and (B) MMP-9 in IDC patients and healthy individuals. ROC, receiver operating characteristic; VEGF, vascular endothelial growth factor; MMP-9, matrix metalloproteinase-9; IDC, infiltrative ductal carcinoma.

Receiver operating characteristic (ROC) curve analysis (Fig. 4) revealed the area under the ROC curve for VEGF was 0.788 (95% confidence interval, 0.711–0.864), whereas for MMP-9, the area under the ROC curve was 0.861 (95% confidence interval, 0.806–0.916). Thus, serum VEGF and MMP-9 levels may be used as markers for the diagnosis of breast IDC.

## Discussion

Angiogenesis is known to be closely associated with carcinogenesis and tumor development. Neovascularization provides oxygen and nutrients to tumor cells, and endothelial cells can promote cell growth in breast carcinoma via paracrine signaling and the production of carcinogenesis promoters (8). Neovascularization of tumors is the result of the dynamic equilibrium between two groups of factors that promote and inhibit vascularization. VEGF is potent in promoting vascularization (9,10). It is an endothelial cell specific mitogen that markedly increases vascular permeability by binding to vascular endothelial growth factor receptor (VEGFR)-2. When VEGF binds to VEGFR-3, VEGF activates the Ras/mitogen-activated protein kinase (MAPK) and related adhesion focal tyrosine kinase signaling transduction pathways, which promotes lymphangiogenesis of the tumor (11–13). MMP-9 promotes vascularization of the tumor by upregulating the bioavailability of VEGF. In addition, MMP-9 can regulate the stability and permeability of newly formed tumor vessels (14).

In the present study, the levels of VEGF and MMP-9 were compared among IDC patients, fibroadenoma patients and healthy adults. The results demonstrated that the levels of VEGF and MMP-9 in IDC patients were significantly higher than those in other two groups. In addition, the levels in the fibroadenoma patients were slightly higher than those in the healthy adults, although there was no statistically significant difference. In IDC patients, the levels of VEGF and MMP-9 were associated with their TNM-staging. Significantly higher levels of VEGF and MMP-9 were observed in patients classified as TNM stage III and IV as compared with patients classified as TNM stage I and II. A previous study demonstrated that VEGF levels increase in parallel with TNM-staging (15). In addition, MMP-9 levels are similar in triple negative breast cancer (TNBC) and in estrogen-dependent breast cancer (16). These observations are consistent with the results of the present study.

In the current study, a significant association was observed between the tumor size and the levels of VEGF and MMP-9, with levels increasing in parallel with tumor size. Goldhirsch *et al* (17) reported that a tumor size of >2 cm indicated poor prognosis in breast cancer patients. Tumor size had been regarded as an independent factor for prognosis of breast cancer. Generally, larger tumors indicate poorer prognosis when regional lymph node metastasis and distant metastasis are excluded. Rakha *et al* (18) also reported that tumor size played an important role in indicating the prognosis and particularly in guiding treatment in TNBC (19). Thus, the levels of VEGF and MMP-9, as a factor in parallel with tumor size, can also indicate the prognosis in breast cancer. To exclude the influence of chemotherapeutic drugs, serum was collected postoperatively and prior to the start of chemotherapy. The levels of VEGF and MMP-9 were found to decrease as a result of tumor cytoreduction, thus, it was hypothesized that the levels of VEGF and MMP-9 may reflect the control of breast cancer.

VEGF can promote the proliferation of epithelial cells and the permeability of vessels, which facilitates the invasion and metastasis of tumors. MMP-9 can degrade the basement membrane, which also leads to metastasis. The incidence of lymph node metastasis in patients with high levels of VEGF or MMP-9 has been reported to be higher compared with patients with low levels of VEGF and MMP-9. In addition, the incidence is higher in patients with high levels of both factors as compared with a high level of a single factor (15). In the present study, the levels of VEGF and MMP-9 were shown to be significantly higher in patients with lymph node metastasis than in patients without lymph node metastasis. Thus, we hypothesized that the levels of VEGF and MMP-9 may be used as risk factors in predicting lymph node metastasis. In addition, the levels of VEGF and MMP-9 were significantly higher in IDC patients than in the fibroadenoma patients and healthy adults, and the level of MMP-9 exhibited a positive correlation with the level of VEGF. These observations indicate that VEGF induces high expression levels of MMP-9 by the continuous activation of the MAPK pathway. As a result, induced EMT of the tumor cells promotes advancement, invasion and metastasis. However, VEGF may activate mitosis and facilitate the survival and proliferation of endothelial cells in the lymph vessels. Thus, the increase in lymphatic vessel density at the periphery of the tumor may also contribute to invasion and lymph node metastasis in breast cancer.

The positive correlation between VEGF and MMP-9, as shown in the present study, was consistent with the results of previous studies. Riedel *et al* (20) reported that a synergy existed between VEGF and MMP-9 levels and that the two factors can be used as biomarkers to indicate the neovascularization, invasion and metastasis of tumors. Riedel *et al* (21) reported a correlation between VEGF and MMP-9 levels in the neovascularization of head and neck squamous cell carcinoma. Zucker *et al* (22) reported that VEGF can promote the secretion of MMP-9 and increase the activity (23). Thus, combined detection of VEGF and MMP-9 exhibits a certain value in predicting the invasion and metastasis of tumors.

Carcinogenesis in breast cancer is a multi-factor process, and invasion and metastasis involves multiple steps, thus, is very complicated (24). VEGF and MMP-9 are important cytokines that participate in the regulation of the aforementioned processes. Through combined detection of VEGF and MMP-9, the present study identified that the levels were correlated with TNM-staging, primary tumor size and lymph node metastasis in breast cancer. A correlation was also observed between the two factors. Therefore, combined detection can play a supporting role in determining TNM-staging and lymph node metastasis in IDC patients.

In the present study, a lipid chip-based method was used to detect the serum levels of VEGF and MMP-9. Compared with pathological methods and ELISA, the lipid chip-based method has higher sensitivity and accuracy (accurate to 0.01 pg/ml) (25). The liquid environment is more suitable for maintaining the formation of biomacromolecules and for the reactions between probes and substrates. The lipid chip-based method simply requires blood samples, thus, this method has a number of advantages, including low cost, small wounds, easy surgery and good repeatability (26).

The present study has certain limitations. Platelets have been shown to release VEGF into the serum, thus, the level of VEGF may be influenced by the amount and the activity of platelets. Therefore, detecting the plasma levels of VEGF and MMP-9, rather than the serum levels, may be more accurate (27). Further research with more patients observed over a longer follow-up period is required to confirm the association between VEGF, MMP-9 and the prognosis of IDC patients.

## Figures and Tables

**Table I:** Serum VEGF and MMP-9 levels in IDC and breast fibroadenoma patients and healthy adults.

Group	VEGF (pg/ml)	MMP-9 (ng/ml)
IDC	152.76 (<0.025-1044)	945.09 (63-5112)
Preoperative (n=301)	180.89 (1-926)	903.92 (115-3495)
Postoperative (n=118)	135.26 (6-676)	680.36 (67-2348)
Fibroadenoma (n=83)	29.86 (7-84)	563.59 (135-895)
Healthy adults (n=40)	52.45 (<0.025-215)	267.33 (19-916)

VEGF, vascular endothelial growth factor; MMP-9, matrix metalloproteinase-9; IDC, infiltrative ductal carcinoma.

**Table II:** Association between serum MMP-9 and VEGF levels with clinicopathological characteristics of IDC patients.

Characteristics	Cases (n)	VEGF (pg/ml)	P-value	MMP-9 (ng/ml)	P-value
Tumor size (cm)			<0.001		<0.001
<2	133	96.55 (1-345)		524.71 (63-2553)	
<5≥2	114	158.61 (<0.025-1044)		1051.40 (101-5112)	
≥5	54	278.89 (13-926)		1756.00 (392-4786)	
Clinical stage (UICC 7^th^)			<0.001		<0.001
I	125	86.12 (0-488)		413.05 (63-833)	
II	58	106.14 (10-281)		682.33 (125-1377)	
III	98	207.2 (13-1044)		1435.35 (533-3041)	
IV	20	295.55 (52-926)		2630.05 (1035-5112)	
Lymph node metastasis			0.001		<0.001
Negative	166	93.62 (0-1044)		475.77 (63-1377)	
Positive	127	231.07 (13-926)		1566.80 (445-5112)	
Missing	8	136.88 (1-319)		813.88 (238-2337)	
Age (years)			0.53		0.104
<35	21	122.38 (10-345)		783.38 (125-2553)	
<50≥35	116	154.22 (13-756)		1006.11 (82-4786)	
≥50	164	155.62 (0-1044)		922.63 (63-5112)	

MMP-9, matrix metalloproteinase-9; VEGF, vascular endothelial growth factor; IDC, infiltrative ductal carcinoma; UICC, Union for International Cancer Control.
